# A qualitative study of parental views of HPV vaccination in Ireland

**DOI:** 10.1080/13814788.2020.1851677

**Published:** 2021-01-26

**Authors:** Stephanie Creed, Elaine Walsh, Tony Foley

**Affiliations:** a Mercy University Hospital, Cork, Ireland; b Department of General Practice, University College Cork, Cork, Ireland

**Keywords:** HPV, vaccination, parental, Irish, qualitative

## Abstract

**Background:**

Despite significant evidence supporting the Human Papillomavirus (HPV) vaccine in the prevention of cervical cancer, uptake of this vaccine is below target in many countries. HPV uptake in Ireland has declined from 87% in 2014–15 to 51% in 2016–17 and currently remains suboptimal at 64.1% in 2017–18.

**Objectives:**

This study aimed to explore parental views of the HPV vaccine; elucidate specific concerns relating to this vaccine and to identify relevant influences on the decision to vaccinate against HPV to inform strategies to optimise uptake.

**Methods:**

An in-depth qualitative study, using semi-structured interviews was conducted among parents of 11–13-year-old girls (*n* = 18) who had not yet been offered the HPV vaccine. Convenience sampling was used. Interviews, conducted in the Republic of Ireland over six-months in 2018, were audio-recorded, transcribed, and analysed by thematic analysis.

**Results:**

Eighteen interviews were conducted (14 female and 4 male participants). Parents favoured HPV vaccination to protect their daughters and prevent disease. Barriers to vaccination included; the fear of long-term side effects, lack of knowledge and the risk versus benefit ratio. General practitioners (GPs) were identified as having a strong influence over parental vaccination decisions, as did media reports and the recent cervical screening programme controversy in Ireland.

**Conclusion:**

This study suggests that significant parental concerns remain to the HPV vaccine. More comprehensive information on the research surrounding this vaccine’s safety profile is required. GP’s may play a pivotal role in HPV vaccination going forward.

KEY MESSAGES

- Though parents demonstrate good knowledge of the protective effect of the HPV vaccine, significant concerns remain surrounding side effects reported in the media.
- Further education to disprove reported associated side effects is required.
- Overall parents were in favour of HPV vaccination to reduce the risk of cervical cancer.

## Introduction

Human Papillomavirus (HPV) is a sexually transmitted viral infection that is spread *via* skin-to-skin contact. HPV has a high prevalence worldwide, with almost all of the sexually active population becoming infected at some point in their lives [1,2]. Chronic HPV infection is the most common underlying cause of cervical cancer as well as being strongly linked with oropharyngeal, anal, vulval and penile cancers, and genital warts. Two particular strains of HPV, type 16 and 18 are accountable for 70% of cervical cancers and precancerous cervical lesions [3]. Each year in Ireland, an average of 264 women are diagnosed with cervical cancer and approximately 90 of these women subsequently die from the disease [1,4].

The HPV vaccine has been shown to significantly reduce the occurrence of high-grade cervical intraepithelial neoplasia, the precursor of cervical cancer [3,5,6]. Ireland introduced the vaccine into the National Immunisation Programme in 2010, offering the vaccine to all females upon entering second-level education (12–13 years of age) and was initially well-received [7]. Since September 2019, Irish males have also been offered the HPV vaccine upon entering second level education. The vaccination target of 80% was exceeded in the years 2011–12 through to 2014–15 [8–11]. However, the percentage uptake in Ireland significantly declined from 87% in 2014–15, to 72% in 2015–16 and more recently 51% in 2016–17 [8,12,13]. The specific reasons behind this drop in vaccination rates is unclear, however, the timing coincides with the establishment of anti-HPV vaccine lobby groups, which raised concerns over the safety profile of the vaccine in Ireland [14].

The HPV Vaccine Alliance was formed in 2017 to restore HPV vaccination rates [15]. In the same year, the Irish Health Service Executive (HSE) launched a large-scale media campaign promoting HPV vaccination [15]. These efforts led to an improvement in vaccination rate of 64.1% in 2017–18, but uptake remains suboptimal [16].

Concerning trends of HPV vaccine uptake have also been noted in other European countries. Denmark successfully introduced the HPV vaccine to their immunisation programme in 2009 with impressive uptake rates of 90%, however by 2014 this had diminished to 54%. The decline also followed an increase in negative public attention surrounding suspected adverse effects from the vaccine [17]. France has one of the lowest uptakes across Europe, fluctuating between 15 and 30% for full course completion [18]. Most of the other European countries, however, have not seen the same sudden unprecedented decline in HPV vaccine uptake; instead, their rates have remained relatively stable since vaccine introduction [19–21].

There is currently lack of published research on parental views regarding HPV vaccination in Ireland. While it has been postulated that the decline in HPV vaccination may be linked to the concerns of lobby groups regarding vaccine safety, a causal relationship has not been established, however. The present study seeks to address the gap identified in the published literature and to provide insights that may help develop strategies to improve HPV vaccination uptake.

## Methods

### Design

A qualitative approach was adopted to obtain an in-depth understanding of issues pertaining to HPV vaccination. Semi-structured face-to-face interviews were conducted. A topic guide for interviews was developed based on a review of the existing literature and included considerations specific to the Irish context as determined by the research team (SC – female, a final year medical student, TF – male, an academic GP with experience in qualitative research, EW – female, an academic GP with experience in qualitative research). Two pilot interviews were conducted, resulting in minor adaptations to the topic guide. Prompts in the topic guide included general opinions on vaccines, knowledge of HPV and HPV vaccination, specific concerns regarding the HPV vaccine and potential methods of addressing concerns (Box 1). In addition, the current HPV vaccine information leaflet published by the HSE was presented to the interviewees for their review and comments [22].

### Setting

The study was conducted in the Republic of Ireland over six-months. Participants were selected from a large GP practice in Co. Cork with 7 General Practitioners (GPs) and caring for circa 13,000 registered patients. This practice was selected, as it is nationally representative in terms of patients’ socioeconomic status (SES) and geographical location. Medical card status (means-tested national public health insurance system entitling the holder to free access to healthcare) was used as a proxy measure for socioeconomic (SES). Geographical location was determined as living in an urban or rural area.

### Sampling

Inclusion criteria for this study were parents of female patients aged 11–13 years, registered to the practice, who had not yet been offered the HPV vaccine. Inclusion criteria were applied by practice GPs to all currently registered practice participants; convenience sampling was then used to contact potential participants to inform them of their eligibility to partake. Once the GP had obtained verbal consent, participants were then contacted by the primary author (SC). Further information provided to participants included the participant invitation letter, participant information leaflet and consent form (Supplementary Appendices 1 and 2). Participant recruitment was ongoing in line with data analysis.

### Data collection

All interviews were carried out by the primary author (SC), with the interviewee in a private room in the GP practice, except one interview, which took place in the participant’s home. SC completed a ten-credit research module, which included qualitative research methods and interview skills and techniques. Written consent was obtained from each participant. Interviews were audio recorded and transcribed verbatim. Field notes were made during and post interviews. Transcripts were subsequently sent to participants for review and no further edits were required.

Interviews began in March 2018 and were carried out until data saturation was reached in August 2018. While there is no one definitive test for data saturation, the method described by Francis et al., was employed for this study [23]. An initial sample size of ten was set and data saturation was tested, by conducting subsequent interviews. As new themes emerged from the initial three subsequent interviews, three further interviews were conducted. Again new themes emerged, so a further two interviews were conducted after which data saturation was reached. This was confirmed by the absence of any new themes emerging.

### Data analysis

In terms of methodological orientation an inductive approach was adopted, data were analysed iteratively and thematic analysis as described by Braun and Clarke was conducted [23]. SC and TF conducted dual independent coding of the first three transcripts of interviews. Transcripts were read and initial codes were generated and discussed at a research meeting, and a coding system agreed (Table 1). All subsequent interviews were analysed by one of the researchers (SC). For every three interviews, one was selected at random for dual independent coding analysis by a second researcher (TF). NVivo Software Version 11 was used for data management. The consolidated criteria for reporting qualitative research (COREQ) statement was used to inform reporting of the findings (Supplementary Appendix 5).

**Table 1.** Themes, subthemes and codes.

Theme	Subtheme	Codes
Knowledge	HPV virus HPV vaccine	Associated diseases / likelihood of infection / prevalence / prevention / immune response / ingredients
Benefit of vaccination	Protection Disease prevention	Modern medicine / life saving / other vaccines
Issues of concern	Side-effects Risk versus benefit Lack of information	Fear of unknown / unreported side effects / MMR autism link / parental peer pressure / induce cancer / pharmaceutical lobbying / too young
Influencing factors	Media GP Cervical screening controversy	Scaremongering / lobby groups / trust in medicine / lack of trust in government or health service

### Ethics

Ethical approval was sought from the Clinical Research Ethics Committee (CREC) of University College Cork on 17th of November 2017 and granted prior to initiating the study (Supplementary Appendix 3)

## Results

In total, 27 parents were contacted by the GP. Of the 27 parents, five did not respond to further contact, four declined (one due to lack of interest, three due to lack of availability), 18 agreed to participate and were interviewed. Participant demographics are shown in Table 2. The mean age of parents interviewed was 45.3 years (SD 4.3 years). Mean interview duration was 41 min (SD 10.8 min).

**Table 2.** Participant demographics.

Demographics – Participants *n* = 18	Participants *n* (%)
Gender	
Male	4 (22)
Female	14 (78)
Occupation	
Professional	9 (50)
Skilled	5 (28)
Unskilled	4 (22)
Age	
30–39	1 (6)
40–45	13 (72)
46–50	2 (11)
51+	2 (11)
Daughters Age	
11	7 (39)
12	9 (50)
13	2 (11)
Daughters Nationality	
Irish	13 (72)
Mixed Irish	2 (11)
Non-Irish	3 (17)
Socioeconomic Status	
Medical Card	3 (17)
Private	15 (83)
Previous HPV Vaccine Decision (older daughter)	
Yes	2 (50)
No	2 (50)
Parent is a Health Care Professional	
Yes	3 (17)
No	15 (83)

### Knowledge

Parents demonstrated varying knowledge regarding different aspects of HPV infection and vaccination. Good knowledge of mode of transmission, presence of a range of subtypes and exposure to the virus resulting in the potential to lead to cervical cancer was displayed. However, misconceptions around HPV infection and vaccination were also reported. While parents were aware an optimal age for vaccination exists, they were unsure as to the reason why. A minority of parents managed to correctly link sexual transmission to the importance of vaccination prior to sexual contact. Some incorrectly proposed the developmental stage of the body as an explanation and the majority simply did not know why. Parents also vastly underestimated the prevalence of HPV infection amongst a sexually active population;

*It’s not the like the usual ones you hear about like chlamydia or gonorrhea* (P14); *I would say 1% of population* (P8).

#### Benefit of vaccination

##### Protection

Health protection was the primary reported reason as to why parents would choose to vaccinate against HPV;

*To protect your child, no matter what, in any way possible* (P12).

Despite parents not openly referring to their daughter’s future sexual activity, many did allude to the concept that their daughter would, go on to *‘live a normal life’* (P6). The recognition of this for parents was the basis of their reason to *‘protect her now, while we still can’* (P8). The majority of participants mentioned protection from cervical cancer.

##### Disease prevention

The fear of cancer was a strong driving force amongst parents*;*

*Cancer is the number one killer in the western world so I think whatever chance you have to prevent it, you should* (P10).

Parents viewed the HPV vaccine as a method of preventing cancer;

*Sure we all know prevention is better than cure, I’m sure abstinence is even better again but that’s a perfect world and we have to deal with the practicalities of life* (P6).

No parents mentioned the prevention of sexually transmitted infections.

#### Issues of concern

##### Side effects

Some parents expressed *‘slight concerns’* to vaccines in general; in particular parents reported nervousness over

*How much they (children) get into their little systems* (P14).

Parents mentioned the MMR and alleged autism link, most dismissing the possible association, while a minority admitted the fear is still present in their minds. The risk of side effects was reported as a reason for parents choosing not to vaccinate:

*I have those poor parents’ voices in my head saying, ‘My child just isn’t the same*’ (P15).

Immediate vaccine reaction was also a concern, however, the parents who voiced concerns regarding side effects admitted their main fears to be associated with long-term effects, in particular *‘chronic fatigue syndrome’* (P13). Parents cited reports from other parents alleging their daughters to have been, adversely affected by the HPV vaccine;

*The HSE (Health Service Executive) are swearing that there are no side effects but then you have parents swearing there are* (P12).

##### Risk versus benefit

The risk versus benefit ratio was another aspect of parental doubt;

*You just don’t know what to do, I mean it’s all a risk* (P13).

The undecided parents spent a large proportion of the interview debating the pros and cons of vaccination and contemplating other strategies to protect their daughter, such as education, *‘if we tell her the risks, she might be more careful’* (P12) or delay vaccination until *‘she is more able to understand’* (P12). Overall, the undecided parents viewed the risks of HPV vaccination to be as great as the benefit leaving them feeling *‘stuck between a rock and a hard place’* (P13).

##### Lack of information

Lack of information was a major source of concern for parents regarding HPV vaccination,

*I just feel I’m not getting all the information* (P3).

In particular, parents felt the reports of these adverse long-term effects had *‘never been fully addressed’* (P14) or *‘brushed under the carpet’* (P12) by the HSE. One parent commented:

*I don’t think anybody ever put a line under it and said, right – that’s what happened there* (P14).

Parents were content with the information provided in the HPV leaflet. However, the main criticisms were that some of the answers left room for more questions. For example,

*Why is this the optimum age?* (P4), *Why two doses at 12 and three doses at 15 or older?* (P3) and *It says no long term side effects – so what happened to those girls then?* (P15).

#### Influencing factors

##### Media

For most parents, adverse media reports gave rise to questioning of the decision to vaccinate;

*You hear a mother crying on the radio as you’re drinking your coffee and you just feel for her first of all, and then think, will I be the mother crying on the radio next year?* (P15).

Parents offered the opinion that information presented in the media may be biased towards extreme viewpoints;

*I don’t enjoy those shows as feel they are unbalanced and hysterical* (P1).

However, parents still questioned why adverse effects are so frequently reported;

*it does beg the question why are there so many reports* (P3).

Radio talk shows were the predominant media source of influence, followed by online and print articles, social media and television. Parents perceived the attitude towards the HPV vaccine from all media sources to be negative overall. However, they reported recently noting the emergence of positive information. One parent described the effect a recent article in the newspaper had on her decision. This mother admitted to becoming quite anxious after listening to radio talk shows on the topic and considered delaying vaccination for her daughter but reading this *‘powerful piece by Dr. Ciara Kelly [GP and radio presenter] made me stop in my tracks and think what would I be waiting for’* (P1).

##### Gp

Parents reported the opinion of their GP as the strongest positive influence on the decision to vaccinate. Most parents stated they would turn to their GP if in doubt, in preference to any other health care professional. Parents reported relief and comfort associated with reassurance from a GP regarding the decision to vaccinate;

*it’s the person you rely on the most and trust their opinion* (P6).

Some parents expressed their intention to make an appointment to discuss HPV vaccination and the majority stated that an opportunity to meet with a GP to ask questions would be of benefit.

##### Cervical screening controversy

The Irish cervical screening controversy was reported in the media in April 2018, as described in Box 2. Six of the 18 interviews in this study were carried out prior to April 2018, meaning this topic did not feature in their interviews. The dominant emerging theme amongst the remaining 12 participants was the heightened awareness of cervical cancer;

Box 1 Semi Structured Interview Guide.

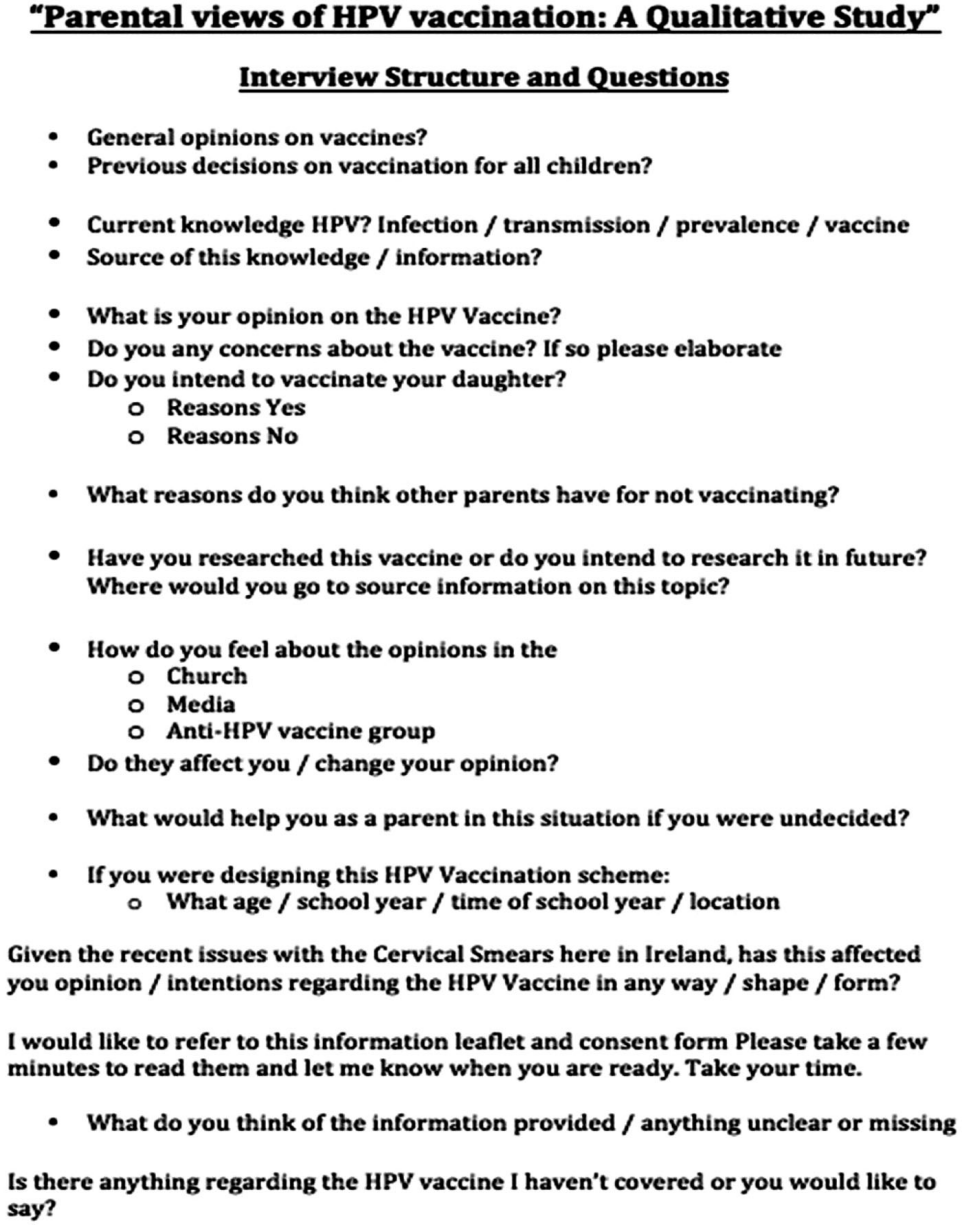

Box 2  

The Irish cervical screening controversy was reported in the media in April 2018. This controversy arose after Vicky Phelan, a 43-year old mother, diagnosed with cervical cancer, settled a High Court case against a US laboratory. Cervical Check, the Irish national screening programme, subcontracted this laboratory to analyse cervical smear samples. In 2014, the year Mrs. Phelan was diagnosed with cervical cancer, her sample was audited and found to have been read incorrectly, however, Mrs. Phelan was not informed of these findings until September 2017, despite the US laboratory, Cervical Check and her doctors being aware of the results much earlier [29]. As Ms. Phelan’s story unfolded, 16 other Irish women were notified they also had misread cervical smears, at this point the majority of these women were either undergoing treatment for cervical cancer or had unfortunately already died from the disease [30].

*The population are a hell of a lot more aware of the topic* (P8) and *I would never have considered that so many women that young could get cancer* (P6).

The strong impact this scandal had on parents resulted in two viewpoints. On the one hand, parents were grateful to have a vaccine to prevent, not only these events recurring but the actual root of the problem and predicted an improvement in the uptake of HPV vaccination;

*I’d say it makes a stronger case for HPV Vaccination, as it puts it into stark reality, makes you realise how fantastic it is to have a vaccine for this disease* (P11).

On the other hand, parents also commented on how the public viewed this situation as yet another failing of the HSE;

*I think its possibly changed people’s opinion on the HSE rather than HPV – I think it’s made people even more conscious that they have lied about this so there is a possibility they are lying about side effects of HPV also* (P12).

## Discussion

### Main findings

To the authors’ knowledge this is the first study evaluating Irish parental views and decision-making regarding HPV vaccination. While parents displayed considerable knowledge regarding HPV, gaps in knowledge were also identified; namely the under-estimation of the prevalence of HPV infection and the specific reasons for vaccination timing. Parents supported the principle of HPV vaccination and expressed a desire to protect their daughter and prevent disease. Fear of inflicting harm was a significant concern expressed however, with interviewees citing reports of adverse effects in the media and the historical MMR and autism controversy as factors negatively influencing their decision to vaccinate. Additional concerns voiced were lack sources of detailed information pertaining to the vaccine and lack of evidence of long-term efficacy. Parents viewed the recent cervical screening controversy as having both positive and negative impacts on HPV vaccination; the scandal highlighted the prevalence of cervical cancer but also undermined the credibility of the HSE and hence their campaign to promote HPV vaccination. Parents expressed overall trust in their GP and identified their GP as the greatest positive influence on their decision to vaccinate.

### Strengths and limitations

To the authors’ knowledge, this is the first study evaluating Irish parental views and decision-making regarding HPV vaccination. The study is timely, given current suboptimal vaccination rates and the recent cervical cancer screening controversy in Ireland. A further strength of the study is the in-depth exploration of a complex and sensitive topic through conducting individual face-to-face interviews. Interviews provided a safe non-judgmental environment and avoided participant-to-participant bias. External validation of the findings was enhanced by seeking feedback from interviewees on the transcripts of their interviews.

Limitations of this study include (1) Sampling technique, which may have introduced selection bias both from the GP and the participant e.g. parents with a more positive attitude towards vaccination may have been more likely to participate. Purposive sampling was attempted at the outset of the study to achieve adequate demographic representation. However, as there was an ethical need to involve the patient’s GP in the recruitment process, in addition to some patients declining to participate, pragmatic constraints arose necessitating the use of convenience sampling. (2) Social desirability bias as the interviewees may have been influenced by awareness that the interviewer is affiliated with the medical profession. (3) Whilst acknowledging the limitations of recruiting patients from a single GP practice, the practice did, however, provide adequate representation of the Irish population in terms of patient demographics, and finally, given the nature of qualitative studies, (4) findings may be deductive but are not necessarily conclusive.

### Comparison with existing literature

A recent systematic review, which included seven European studies, identified the fear of adverse effects associated with the HPV vaccine as a global concern. Side effects reported being most feared included, ‘paralysis, infertility, impaired development, increased risk of HPV infection and cancer, allergy and autism’ [24]’. In contrast, our findings indicate chronic fatigue syndrome to be the most feared adverse effect. While evidence does not support this association of an increased risk of chronic fatigue syndrome with HPV vaccination, this safety concern exists among Irish parents, perhaps attributable to the message from anti-vaccine lobby groups [6,14]. Another European systematic review by Lopez et al., also found safety concerns to be the main barrier to HPV vaccination, however, this was closely followed by fear of encouraging premature sexual activity, a theme that interestingly did not feature in our study [25]. This review included studies published in 16 European countries over 11-years (2006–2017) and concluded that population relevant information for informed decision making on HPV vaccination is required [25]. Our findings support these recommendations.

The essential role of healthcare professionals in reinforcing the importance of HPV vaccination by reiterating rationale behind vaccine recommendations and addressing parental concerns directly has been reported widely in the literature [24]. Our findings indicate that parents identify their GP specifically as their main trusted healthcare advisor on this topic [26]. In light of this GPs must be up-to-date on HPV vaccination and are proactive in instigating discussions and answering questions regarding HPV vaccination.

Our study found that the media was a major source of information for parents when deciding on HPV vaccination. Previous research has found an over-reporting of negative effects of the HPV vaccine in the media and that comprehensive information on the vaccine, HPV, and cervical cancer continues to be missing from media coverage [27].

### Implications for research and practice

The findings of this study suggest that a distorted understanding of the risks and benefits may negatively impact uptake and efficacy of HPV vaccination in Ireland. Further clear and population-specific information needs to be provided to parents, explaining the reasons behind vaccination timing and more importantly displaying the results of high-quality studies disproving the reported adverse effects associated with this vaccine. Although 20 years have passed since the publication and retraction of the controversial study linking the MMR vaccine to autism, this study suggests that vaccine concerns remain. The potential to impact the uptake of all vaccines exists and further measures may need to be employed to address this issue.

This study identified the GP as the single most important positive influence on parental HPV vaccination decision. In light of the fact that results from qualitative studies are not conclusive, further research and consideration should be given as to what role the GP may play in terms of HPV vaccination going forward. Linking GPs with local schools to promote HPV vaccination and to address parental concerns has the potential to impact vaccine uptake positively. Furthermore, the option of receiving the HPV vaccine at the child’s GP practice instead of school could be considered.

A targeted response to the cervical check controversy has the potential, not only to improve screening, but also to highlight the importance of prevention. One young woman, not involved in the controversy but diagnosed with terminal cervical cancer, dedicated her last few months of life to HPV vaccination promotion. Laura Brennan became the face of HPV vaccination urging parents to protect their daughters from this preventable disease. Ms. Brennan passed away in March 2019 and her family has vowed to continue her campaign and not let her death be in vain [28]. Harnessing the momentum of this initiative may assist the HSE in regaining public trust.

## Conclusion

This study suggests that significant parental concern remains surrounding HPV vaccination. Parents require further comprehensive and transparent information to disprove reported associated side effects. GPs may play a pivotal role in the restoration of HPV vaccination rates if adequately supported. While valuable lessons must be learned from the cervical cancer screening controversy, it is also imperative to take advantage of the awareness of cervical cancer that has been raised. By offering parents targeted, clear information and by involving the GP as their trusted healthcare professional the uptake of HPV vaccination can continue to rise.

## Supplementary Material

Supplemental Material
