# Supplementary material for: A qualitative study of parental views of HPV vaccination in Ireland
Source: Eur J Gen Pract. 2021 Jan 26;27(1):1–9. doi: 10.1080/13814788.2020.1851677 (PMC7850332; doi:10.1080/13814788.2020.1851677)

# Appendices

## Appendix 1: Invitation Letter for Participants

#
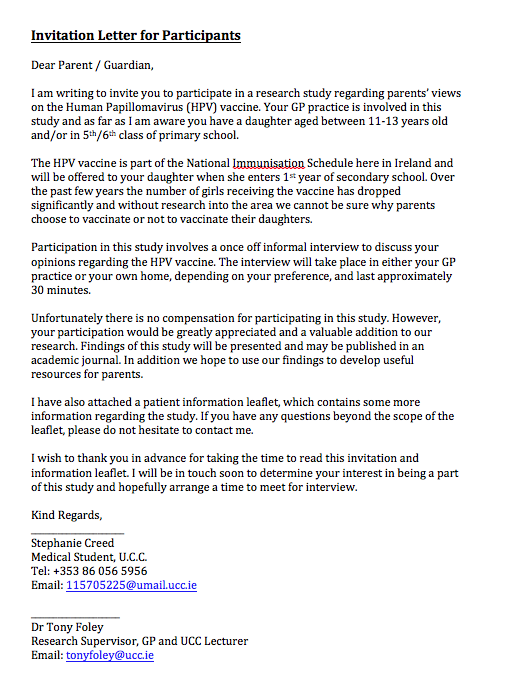


## Appendix 2: Participant Information Leaflet


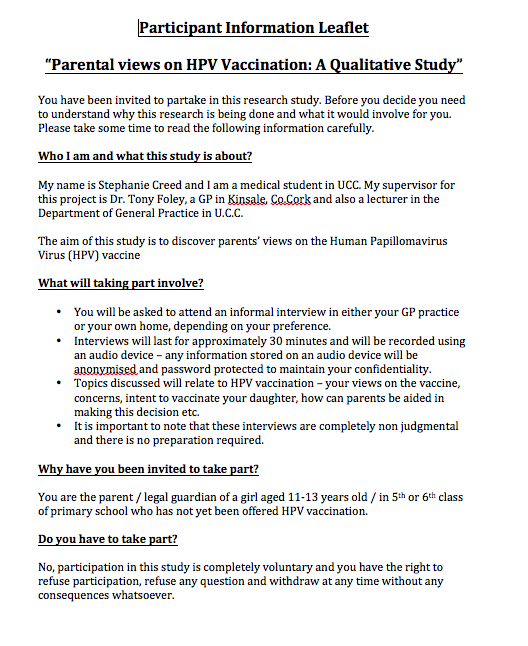


##
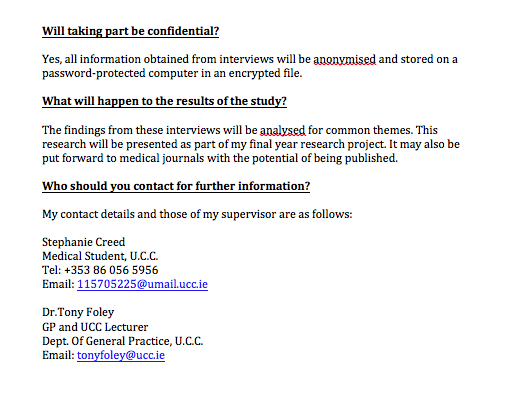

Supplement: Supplemental Material [file IGEN_A_1851677_SM3688.docx]
